# Ultraviolet Radiation and the Slug Transcription Factor Induce Proinflammatory and Immunomodulatory Mediator Expression in Melanocytes

**DOI:** 10.1155/2012/410925

**Published:** 2012-06-13

**Authors:** Stephanie H. Shirley, Elizabeth A. Grimm, Donna F. Kusewitt

**Affiliations:** ^1^Department of Molecular Carcinogenesis, Science Park, The University of Texas MD Anderson Cancer Center, 1808 Park Road 1C, Smithville, TX 78957, USA; ^2^Melanoma Medical Oncology Department, Unit 0362, The University of Texas MD Anderson Cancer Center, Anderson Central (Y8.5325), 1515 Holcombe Boulevard, Houston, TX 77030, USA

## Abstract

Despite extensive investigation, the precise contribution of the ultraviolet radiation (UVR) component of sunlight to melanoma etiology remains unclear. UVR induces keratinocytes to secrete proinflammatory and immunomodulatory mediators that promote inflammation and skin tumor development; expression of the slug transcription factor in keratinocytes is required for maximal production of these mediators. In the present studies we examined the possibility that UVR-exposed melanocytes also produce proinflammatory mediators and that Slug is important in this process. Microarray studies revealed that both UVR exposure and Slug overexpression altered transcription of a variety of proinflammatory mediators by normal human melanocytes; some of these mediators are also known to stimulate melanocyte growth and migration. There was little overlap in the spectra of cytokines produced by the two stimuli. However IL-20 was similarly induced by both stimuli and the NF*κ*B pathway appeared to be important in both circumstances. Further exploration of UVR-induced and Slug-dependent pathways of cytokine induction in melanocytes may reveal novel targets for melanoma therapy.

## 1. Introduction

Although the incidence of melanoma is increasing faster than that of any other neoplasm and the tumor has been studied intensively, the cause of melanoma remains incompletely understood. It is clear from epidemiologic and clinical observations that the ultraviolet (UVR) component of sunlight is an important etiologic factor; however, the precise mechanisms by which UVR drives melanomagenesis are unknown [1–4]. Mutations in oncogenes and tumor suppressor genes have been identified in melanomas, but most of these are not the UVR signature mutations found in nonmelanoma skin cancers [5]. This suggests an indirect role for UVR in melanomagenesis, likely as a tumor promoter [6].

 An important mechanism for tumor promotion is the creation of a tumor microenvironment that fosters tumor cell proliferation and migration while suppressing an effective immune response to the tumor cells. It has been shown that many tumor cell types and their precursors secrete a wide variety of proinflammatory and immunomodulatory substances [7, 8]. For instance, UVR exposure of keratinocytes promotes the production of cytokines, chemokines, and prostaglandins that strongly promote nonmelanoma skin cancer [9]. Our studies in mice have shown that the Slug (Snai2) transcription factor plays a critical role in UVR-induced cutaneous inflammation. Slug knockout mice are highly sunburn resistant, apparently because Slug knockout keratinocytes are unable to secrete appropriate proinflammatory mediators [10].

 Based on our findings in keratinocytes, we examined the possibility that UVR also induces expression of proinflammatory and immunomodulatory mediators by melanocytes. We also investigated a possible role for Slug in modulating UVR induction of these mediators.

## 2. Materials and Methods

### 2.1. Cell Culture

Normal human melanocytes of neonatal origin were obtained from the American Type Tissue Collection (ATCC) and maintained in Dermal Basal Medium supplemented with Melanocyte Growth Kit (ATCC).

### 2.2. UVR Exposure

 Melanocytes were grown to confluence then exposed to 300 J/m^2^ UVR and harvested 24 hours later. UVR was obtained from a bank of Philips TL20 W/12 RS UVB lamps (American Ultraviolet Company). These lights emit wavelengths between 280 and 400 nm, with a peak at 313 nm. The lamps emit approximately 60% UVB wavelengths and 40% UVA wavelengths, with less than 3% of the radiation in the UVC range [11].

### 2.3. Adenoviral Transduction

Melanocytes were grown to 75% confluence then incubated with adenoviral Slug (AdSlug) or a control adenovirus (Ad-CMV-Null, Vector Biolabs) [12]. For transduction, 10 *μ*L of adenoviral stock (viral titer 10^8^ ifu/mL) mixed with one mL of medium was added to each well of a six-well plate. Cells were incubated with the adenovirus for 24 hours before the medium was changed. Cells were harvested 24 hours later, and mRNA was isolated utilizing the RNeasy kit (Qiagen).

### 2.4. RT-QPCR

For mRNA analysis cDNA was generated utilizing the Applied Biosystems High Capacity cDNA RT kit. Following reverse transcription, RT-QPCR was performed utilizing applied biosystems assays on demand for Snai2 and 18S together with TaqMan Universal Master Mix. Amplifications were carried out on an applied biosystem 7900HT real-time PCR analyzer, utilizing the standard curve method for quantification [13].

### 2.5. Western Blotting

 Whole cell lysates in RIPA buffer plus HALT protease inhibitor cocktail (Thermo Scientific) were cleared by centrifuging at 10,000 rpm for 10 minutes at 4°C. A total of 40 *μ*g of protein was loaded on a 4–20% tris-HCL gel. Following SDS-PAGE, protein was transferred to PVDF membrane and probed with antibodies against Slug (Cell Signaling Technology) or GAPDH (Abcam). The secondary antibody utilized was anti-rabbit IgG HRP (GE Healthcare). Primary antibodies were used at 1 : 1000 dilutions, while the secondary antibody concentration was 1 : 5000. Blots were incubated overnight at 4°C with primary antibody and two hours at room temperature with secondary antibodies. Prior to exposure to film, membranes were incubated in Super Signal West ECL (Thermo Fisher Scientific). For quantification, densitometry was performed.

### 2.6. Cytokine Arrays

For cytokine profiling, the PAHS-3803 Human Inflammatory Response and Autoimmunity Cytokine Array (SA Biosciences) was employed. This array quantifies the expression of 370 key genes involved in immune response and inflammation, including genes encoding inflammatory cytokines, chemokines, and their receptors as well as genes involved in cytokine synthesis and metabolism and in cytokine-cytokine receptor interactions. RNA was analyzed to check RNA quality using the Agilent RNA 6000 Nano kit. Only RNA with a RIN value less than 7.5 was employed. For each sample, 1000 ng of RNA was used to synthesize the first strand of cDNA using the high-capacity cDNA RT Kit from Applied Biosystems (ABI), following standard ABI Protocol. The amount of cDNA added to each of the 96-well plates was standardized. The cDNA was mixed with iTAQ SYBR Green Supermix (Bio-Rad), and each plate was run using the ABI Prism 7900 HT instrument using the following protocol: 10 minutes at 95°C for 10 minutes; 40 cycles of 0.15 seconds at 95°C and 60 seconds at 60°C; 15 seconds at 95°C; 15 seconds at 95°C and 15 seconds at 95°C. Analysis of each plate was performed using the SDS 2.3 program from ABI using the same threshold and baseline for all biological samples. Two independent sets of samples were analyzed.

### 2.7. Ingenuity Analysis

Genes with expression levels altered more than two-fold in both replicate arrays were identified. Ingenuity Pathway Analysis software (Ingenuity Systems) was used to construct networks that functionally relate genes with markedly altered expression due to UVR exposure or Slug overexpression. These networks are based on algorithms that reflect the connectivity among different genes. A score is generated that indicates the relevance of any given network to the input genes (http://www.ingenuity.com/).

## 3. Results

### 3.1. UVR Induction of Slug

**Figure 1:**
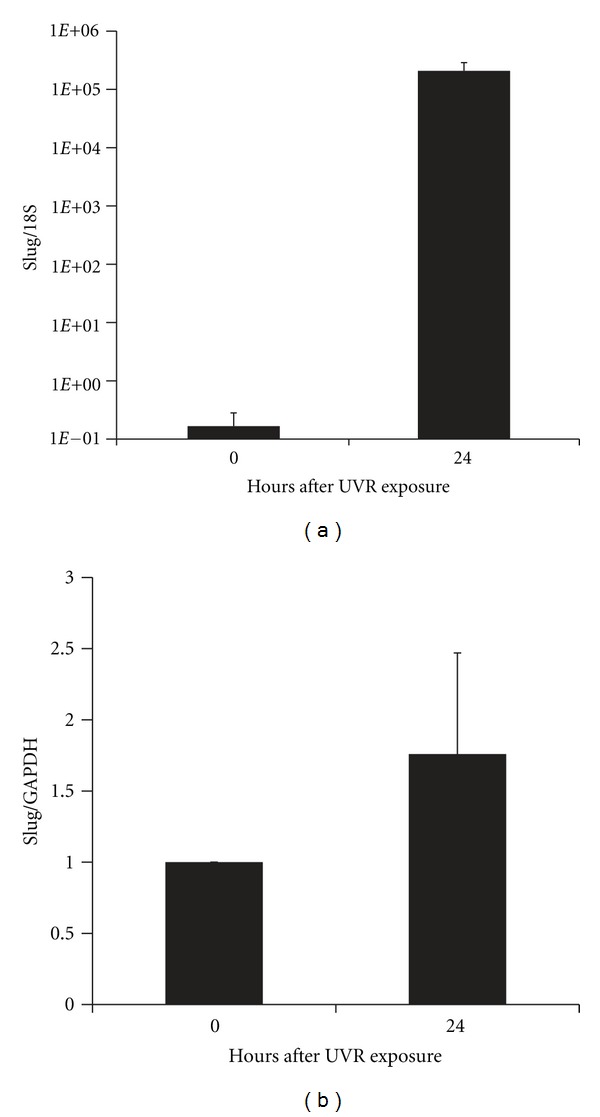
UVR induction of Slug 24 hours following a dose of 300 J/m^2^ UVR. (a) Slug mRNA levels were determined by RT-QPCR in two independent assays, each performed in triplicate. Bars represent the standard error of all six values. (b) Slug protein levels were determined by Western blotting followed by densitometry in three independent assays. Bars represent the standard deviation.

As shown in Figure 1, normal human melanocytes exposed to 300 J/m^2^ UVR showed a marked induction of Slug mRNA expression at 24 hours after exposure. However, Slug protein induction was much less robust, averaging a little less than two-fold at 24 hours. Thus, as in keratinocytes, Slug expression is UVR inducible [14].

### 3.2. UVR and Slug Induction of Proinflammatory and Immunomodulatory Mediators

Two independent comparisons of inflammatory mediator expression in UVR-exposed versus unexposed and in Slug overexpressing versus control normal human melanocytes revealed a number of genes with expression consistently altered two-fold or more.

 Nine genes showed increased expression and six genes showed decreased expression in UVR-exposed versus unexposed melanocytes (Table 1). Genes with the most markedly increased expression included CXCL2, CXCL3, and IL-8. All of these genes, as well as the more moderately increased CXCL1, have been shown to be important both in neutrophil chemotaxis and in melanoma growth [15, 16]. Increased expression of PTGS2 (cyclo-oxygenase-2 (COX-2)) is reminiscent of the situation in nonmelanoma skin cancer. UVR induces PTGS2 in keratinocytes, and the gene is overexpressed in premalignant and malignant nonmelanoma skin lesions [17]. Downregulation of NR3C1 (glucocorticoid receptor) is of interest, as melanoma cells have recently been shown to express this receptor and dexamethasone has an antiproliferative effect on these cells [18]. Other findings were less easily understood. PTAFR (platelet-activating factor receptor) expression was decreased after UVR, although activation of this receptor on melanoma cells has been demonstrated to enhance their proliferation, invasiveness, and metastatic capabilities [19]. Decreased expression of the NF*κ*B transcription factor and the NF*κ*B activator RIPK2 was unexpected, as UVR activates the NF*κ*B pathway in many melanoma cells, and these cells constitutively express both NF*κ*B and an alternative activator NIK [20]. The role of CCL2 production by melanoma cells remains somewhat controversial, as low CCL2 levels appear to attract tumor-associated macrophages, which promote tumor development, while high levels of CCL2 production attract macrophages that cause tumor regression [21]. The relationship of other genes with altered expression to melanomagenesis has not been explored.

AdSlug-transfected melanocytes show markedly enhanced Slug expression [22]. Compared to control melanocytes, Slug-overexpressing melanocytes had more than two-fold increased expression of 13 genes and more than two-fold decreased expression of two genes. The three genes with the most markedly altered expression were CCL18, CXCL10, and IL-4, molecules that are chemotactic for or activate lymphocytes [23, 24]. At the same time, IL-4 has been shown to suppress the growth of melanoma cell lines and enhance their immunogenicity [25], thus enhanced IL-4 expression would not be expected to enhance melanomagenesis. Nor would expression of IFNA4, which has been used in melanoma therapy [26]. Many growth factors stimulate melanocytes proliferation, including FGF2 [27, 28], thus it seems likely that FGF10 may also contribute to melanocyte proliferation. The endothelial protein C receptor (PROCR) is expressed by a variety of tumor cell types, and protein C appears to stimulate tumor cell proliferation, migration, and metastasis [29, 30]; thus, increased expression of PROCR in melanocytes may stimulate them to proliferate and migrate. Interestingly, expression of TACR1 (tachykinin receptor) is downregulated in Slug-overexpressing melanocytes; TACR1 antagonists drive melanoma cell apoptosis, thus Slug overexpression may protect melanocytes from apoptosis [31]. In B cells, BLNK (B-cell linker protein) is important for activation of the NF*κ*B pathway [32]; however, its expression has not been reported in other cell types. Little is known about the role of IL1R2 (IL-1 decoy receptor), IL5RA (IL-5 receptor component), MGLL (monoglyceride lipase), or SERPINF2 in melanomagenesis.

 The only gene with expression similarly increased by both UVR and Slug overexpression was IL-20, a member of the IL-10 family of cytokines. IL-20 plays a poorly understood role in cutaneous inflammation. It is highly expressed by keratinocytes in inflammatory conditions of the skin-like psoriasis and atopic dermatitis; however, the role of the cytokine in keratinocyte biology and immune regulation remains controversial [33, 34]. Expression of the related IL-22 cytokine was also increased in Slug-overexpressing cells. Like IL-20, this gene is clearly important in the interaction between keratinocytes and immune cells, and its expression is enhanced in inflammatory skin conditions [35].

### 3.3. Comparison of UVR and Slug-Related Inflammatory Networks

**Figure 2:**
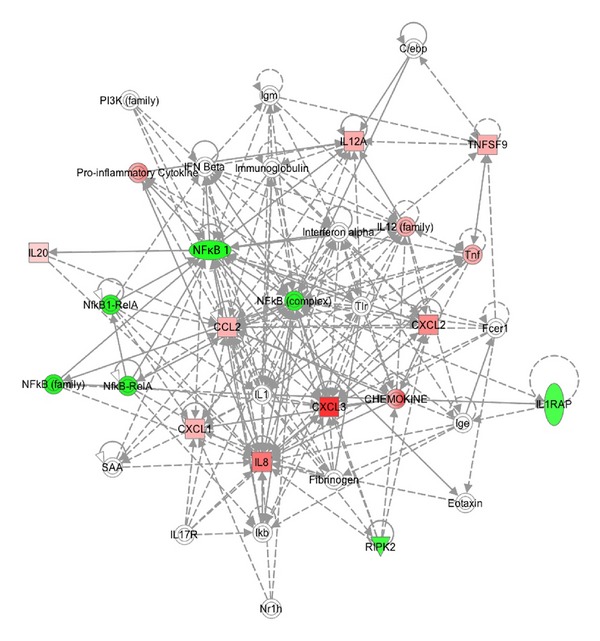
Ingenuity Pathway Analysis of altered expression of proinflammatory genes in normal human melanocytes. Cells were harvested 24 hours following a dose of 300 J/m^2^ UVR. mRNA expression levels were determined using the PAHS-3803 Human Inflammatory Response and Autoimmunity Cytokine Array (SA Biosciences), and genes with expression altered more than two-fold in duplicate assays were included in the Ingenuity Pathway Analysis.

**Figure 3:**
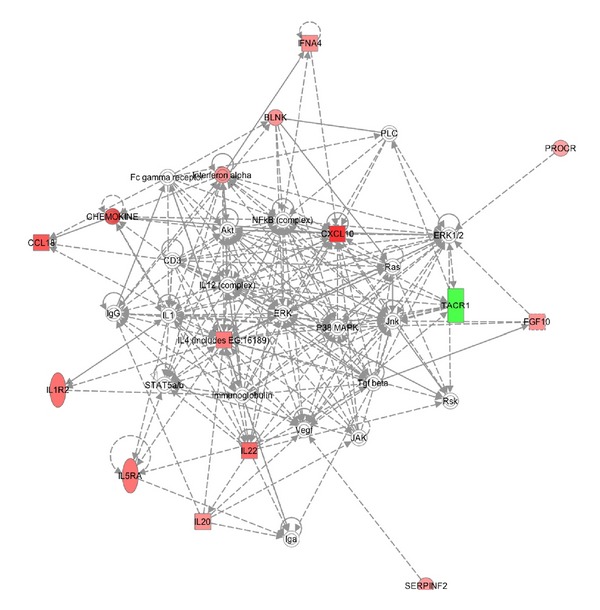
Ingenuity Pathway Analysis of altered expression of proinflammatory genes in normal human melanocytes transduced with Ad-Slug. Expression levels were determined using the PAHS-3803 Human Inflammatory Response and Autoimmunity Cytokine Array (SA Biosciences), and genes with expression altered more than two-fold in duplicate assays were included in the Ingenuity Pathway Analysis.

We investigated interactions among the genes with altered expression in UVR-exposed and in Slug-overexpressing melanocytes using Ingenuity Pathway Analysis (Figures 2 and 3). For each condition, a single network was determined to be highly significantly related to the gene set with altered expression. In both cases, the main functions of input genes were related to cellular movement, immune cell trafficking, and hematological system development. The *P* value, which indicates the probability of an association between genes in the dataset and a given canonical pathway, was ≤10^−30^ for both gene sets. However, because the analysis was based on a limited number of genes preselected for their involvement in inflammation and autoimmunity, this degree of association is perhaps not remarkable. The canonical networks identified accounted for 11/16 genes in the UVR dataset and 13/16 in the Slug dataset. Many of the gene interactions in both datasets were centered around NF*κ*B. The NF*κ*B pathway is constitutively activated in malignant melanoma and is believed to be responsible for persistent expression of chemokines by melanoma cells [20]. For Slug, there was an additional focus centered on ERK and p38 MAPK; this is not surprising, as both Slug induction and NF*κ*B activation may occur via MAPK pathways [20, 36].

## 4. Discussion

UVR makes important contributions to skin carcinogenesis. UVR serves as a complete carcinogen for nonmelanoma skin cancer: it both induces critical mutations in keratinocytes and promotes tumor expansion [37]. One of the ways that UVR promotes the growth of nonmelanoma skin cancers is by stimulating keratinocytes to release proinflammatory mediators, thus creating a local milieu that promotes tumor growth [17, 37]. Indeed, anti-inflammatory compounds like COX-2 inhibitors are highly effective in reducing the progression of UVR-induced nonmelanoma skin cancers in experimental animals [17]. In melanoma, the role of UVR is less clear, as the driving mutations in melanoma do not appear to be due to the direct mutagenic activity of UVR; instead, UVR appears to promote melanoma development by creating a proinflammatory and immunosuppressive environment [4]. To date, studies of UVR-induced cutaneous inflammation have focused on the role of proinflammatory mediators released by keratinocytes; however, our present studies demonstrate that UVR-exposed melanocytes are also able to produce many of the same mediators. Other exogenous stimuli have also been reported to induce the production of cytokines and chemokines by melanocytes and melanoma cells. Lipopolysaccharides can stimulate normal human melanocytes to produce IL-1*β*, TNF*α*, IL-6, IL-8, CCL2, CCl3, and CCL5 [38, 39], and chemotherapy of melanoma-bearing mice results in enhanced expression of CCL5 and the CXCR3 ligands CXCL9, CXCL10, and CXCl11 by tumor cells [40].

 Our previous studies have shown that the Slug transcription factor is an important mediator of UVR-induced inflammation in the skin. Slug knockout mice are highly resistant to UVR-induced skin inflammation because UVR-exposed Slug knockout keratinocytes release fewer proinflammatory mediators than wild type keratinocytes [10]. The role of Slug in the production of inflammatory mediators in melanocytes has not previously been investigated. The present studies demonstrate that both Slug mRNA and protein are UVR inducible and that Slug overexpression in melanocytes stimulates chemokine and cytokine expression. This is particularly interesting, in that Slug is expressed in many melanomas and is believed to contribute to melanoma progression [41]. However, our previous studies have suggested that Slug expression is actually maximal early during melanoma progression [22]. Taken together, our previous and present finding suggest that Slug expression early in melanomagenesis may play an important role in modulating the inflammatory and immune response to melanocytes early during melanomagenesis.

 The spectrum of proinflammatory genes with altered transcription in UVR-exposed versus Slug-overexpressing melanocytes showed little overlap. In fact, the only proinflammatory gene similarly altered in the two conditions was IL-20, which plays a poorly defined, although potentially critical role in cutaneous inflammation [42]. Despite the lack of overlap in gene expression profiles, Ingenuity Pathway Analysis revealed that both patterns of expression centered on NF*κ*B, a transcription factor known to play an extremely important role in melanoma cell production of chemokines [20]. The additional centering of the Slug pathway on ERK and p38 MAPK may be related to the fact that both NF*κ*B and Slug expression are strongly induced by growth factors like EGF, which signals through the MAPK pathway [20, 36]. Another consideration in interpreting the minimal overlap in inflammatory mediator expression induced by Slug and UVR is that we examined only a single time point in what is clearly a transient UVR response. More detailed characterization of the kinetics of proinflammatory gene expression following UVR exposure may reveal more overlap between Slug and UVR-modulated genes.

 Tumor-derived proinflammatory and immunomodulatory molecules, like those stimulated by UVR exposure and Slug expression, clearly influence tumor growth by modulating the inflammatory and immunologic milieu of the tumor as well as by directly stimulating tumor cell proliferation, migration, and invasion [43]. This makes these molecules potentially valuable therapeutic targets in prevention and treatment of melanoma [8, 15, 16, 21]. For example, COX-2 inhibitors have been shown to be cytotoxic and cytostatic for human melanoma cells in vitro and to have potentially beneficial effects in melanoma therapy [44, 45]. This suggests that further exploration of UVR and Slug-induced proinflammatory and immunomodulatory mediators is warranted.

## 5. Conclusion

Both UVR exposure and Slug overexpression induce alterations in the production of proinflammatory and immunomodulatory mediators by normal human melanocytes. These melanocyte-derived cytokines and chemokines may have important effects on the inflammatory milieu, proliferation, and motility of melanocytes and the melanoma cells to which they give rise. Further study will be required to determine the extent to which Slug mediates the effect of UVR on the induction of inflammatory mediators in melanocytes. Additional studies of cytokine and chemokine induction in melanocytes are warranted, in view of the potential therapeutic targets that may be revealed.

## Figures and Tables

**Table 1:** Altered expression of proinflammatory and immunomodulatory mediators by normal human melanocytes.

Treatment	Gene	UVR-exposed/unexposed^a^	Treatment	Gene	Slug-transduced/control^a^
UVR	CCL2	2.50, 6.63	Slug	BLNK	2.99, 2.38
	CXCL1	5.06, 3.80		CCL18	4.77, 3.53
	CXCL2	6.86, 6.53		CXCL10	4.26, 6.03
	CXCL3	10.61, 13.11		FGF10	2.73, 2.77
	IL12A	7.41, 2.21		IFNA4	2.53, 3.13
	IL1RAP	−2.35, −2.07		IFNWP2	2.53, 2.02
	IL20	2.66, 2.79		IL1R2	2.88, 4.21
	IL8	5.92, 10.21		IL20	2.81, 2.70
	NF*κ*B 1	−2.95, −2.61		IL22	2.47, 5.09
	NFX1	−2.54, −2.49		IL4	3.54, 3.04
	NR3C1	−2.80, −2.45		IL5RA	2.49, 4.40
	PTAFR	−3.06, −2.01		MGLL	−2.95, −4.24
	PTGS2	2.93, 2.52		PROCR	2.19, 2.34
	RIPK2	−2.46, −2.25		SERPINF2	2.56, 2.51
	TNFSF9	6.72, 2.86		TACR1	−2.88, −2.92

^a^Values for both replicate assays are shown.
